# Adaptation to serum-free culture of HEK 293T and Huh7.0 cells

**DOI:** 10.1186/1753-6561-8-S4-P259

**Published:** 2014-10-01

**Authors:** Juliana Fontes Beltran Paschoal, Sandra Suarez Patiño, Thaissa Bernardino, Alexandre Rezende, Marcos Lemos, Carlos Augusto Pereira, Soraia Attie Calil Jorge

**Affiliations:** 1 Instituto Butantan, São Paulo, SP, Brazil

## Background

The fetal bovine serum (FBS) is a component of higher value added to the medium, which may cause difficulty in the process of recovery and purification of bioproduct. Considering all these reasons, it is essential to develop a simple cell adaptation process for the culture of cells in serum-free, animal component-free, or protein-free medium conditions [1,2]. The adaptation of cellular lineages to culture FBS or animal protein free medium can allow optimization of cell growth and expression of heterologous genes, facilitating recovery of recombinant proteins expressed in these cells. Mammalian cell growth can be adapted to serum-free media through progressive reductions of serum concentrations [3]. This gradual reduction over time increases the probability of successful adaptation to low-serum or serum-free media^5^by allowing the self-adjustment of the cells to the environment, with the time required depending on the cell line and the composition of the media [4]. Our aim was to establish and to study mammalian cell lines adapted to serum free medium for the expression of recombinant proteins. Thus, we assessed cell growth, nutrient consumption and metabolite production kinetics in the cell culture media tested.

## Methods

In this work, HEK 293T and Huh 7.0 cells were adapted to different serum free media (SFM) using sequential adaptation approach. HEK 293T cells were adapted to 2 different SFM: Hybridoma^®^-SFM and CHO^®^-S-SFM II; while HUH 7. cells were adapted to 4 SFM: Hybridoma^®^-SFM, VP^®^-SFM, CHO^®^-S-SFM II; and Pro293a^®^. Kinetics parameters of adherent cells were analyzed in duplicate for 5 days in 6 well plates with 2 mL of medium. The initial cell concentration in all experiments was 2 × 10^5 ^cells/mL.

## Results and discussion

Established protocols to adapt HEK293T and Huh7 to growing cells in SFM. By changing gradually of medium, we obtained two lines adapted HEK 293T to two SFM and Huh 7.0 to four SFM. The Huh 7.0 cells showed morphology and growth better than HEK293T cells. The cells was evaluated and the maximum growth of the line Huh-7.0 in the medium SFM-Hybridoma and CHO-S-SFM II were 120hrs with 1.0x10^6 ^cells / ml with viability higher than 90%. The maximum concentration HEK 293T cells and viability were similar to Huh 7.0 cells, but those achieved maximum peak of growth in different times. The SFM CHO-S-SFM II obtained maximum growth in 96 hours and Hybridoma-SFM in 72hrs. Metabolic analysis showed differences in the consumption of nutrients and production of metabolites in different lineages.
